# *LincRNA#1* knockout alone does not affect polled phenotype in cattle heterozygous for the celtic *POLLED* allele

**DOI:** 10.1038/s41598-022-11669-9

**Published:** 2022-05-10

**Authors:** Sadie L. Hennig, Bret R. McNabb, Josephine F. Trott, Alison L. Van Eenennaam, James D. Murray

**Affiliations:** 1 Department of Animal Science, University of California-Davis, Davis, CA USA; 2 Department of Population Health and Reproduction, School of Veterinary Medicine, University of California-Davis, Davis, CA USA

**Keywords:** Genetics, Animal biotechnology

## Abstract

A long intergenic non-coding RNA (*lincRNA#1*) is overexpressed in the horn bud region of polled (hornless) bovine fetuses, suggesting a potential role in horn bud suppression. Genome editing was used to test whether the absence of this sequence was associated with the horned phenotype. Two gRNAs with high mutation efficiencies targeting the 5′ and the 3′ regions flanking the *lincRNA#1* sequence were co-injected with Cas9 as ribonucleoprotein complexes into bovine zygotes (n = 121) 6 h post insemination. Of the resulting blastocysts (n = 31), 84% had the expected 3.7 kb deletion; of these embryos with the 3.7 kb deletions, 88% were biallelic knockouts. Thirty-nine presumptive edited 7-day blastocysts were transferred to 13 synchronized recipient cows resulting in ten pregnancies, five with embryos heterozygous for the dominant P_C_ *POLLED* allele at the *POLLED* locus, and five with the recessive pp genotype. Eight (80%) of the resulting fetuses were biallelic *lincRNA#1* knockouts, with the remaining two being mosaic. RT-qPCR analysis was used to confirm the absence of *lincRNA#1* expression in knockout fetuses. Phenotypic and histological analysis of the genotypically (P_C_p) *POLLED*, *lincRNA#1* knockout fetuses revealed similar morphology to non-edited, control polled fetuses, indicating the absence of *lincRNA#1* alone does not result in a horned phenotype.

## Introduction

Horns are naturally occurring in cattle and are useful for protection against predators. Although horn growth occurs largely after birth, differentiation of the horn bud occurs during embryogenesis such that horns are visible at birth. The first sighting of horn bud development in bovine fetuses is observed after 60 days of gestation^1,2^. At this time the horn bud presents as a small yellowish spot on the fetal head. At 3–4 months it appears as a slight indentation and is clearly visible by 5–6 months^1^. Histologically there are noticeable differences in the horn bud region compared to the frontal skin by day 70 of gestation. Dense layering of vacuolated keratinocytes in the epidermis of the horn bud region along with thick nerve bundles differentiate the horn bud region from the frontal skin^1,3^. Horns present an animal welfare concern and horned cattle are also more aggressive and harder to handle, so cows in the US dairy industry are routinely disbudded at birth^4^. To circumvent the requirement for disbudding, much effort has been expended into breeding cattle with a polled phenotype.

Several studies have identified causal mutations for the polled (hornless) phenotype in cattle, but the underlying biological mechanism causing polled remains unclear^3,5–8^. The two known *Bos taurus* allelic variants at the *POLLED* gene associated with the polled phenotype, Friesian P_F_^3,5,7^ and Celtic P_C_^3,7^, are not associated with any known transcript or protein. Carlson et al.^9^ demonstrated that the *POLLED* variant found in beef breeds (P_C_), when introgressed using genome editing to replace the recessive *HORNED* allele (p) in a cell line derived from a horned dairy bull, resulted in two polled bulls when the edited cell line was subsequently used in somatic cell nuclear transfer (SCNT) cloning to produce live calves. One of these was found to be a compound heterozygote P_C_P_C*_ bull where one P_C_ allele was identical to the Celtic P_C_ allele and the second P_C*_ allele had an introgression of the donor plasmid along with the Celtic allele sequences. A follow up study investigating the progeny of this genome edited bull bred to horned Hereford cows demonstrated that both the P_C_ and P_C*_ alleles were inherited as a dominant trait and resulted in six heterozygous P_C_p or P_C*_p calves all with a polled phenotype, further demonstrating the causal role of the P_C_ allele for the polled phenotype^10^.

Allais-Bonnet et al.^3^ identified an additional genetic component potentially associated with the polled phenotype—a long intergenic non-coding RNA (lincRNA) that is annotated as *LOC100848368* and which was termed *lincRNA#1*. LincRNAs are a form of RNA that have very similar characteristics to mRNA, however they do not code for any known proteins. There is still uncertainty as to why lincRNAs exist, but some play important roles in the regulatory functions of vital biological processes^11–13^. The *lincRNA#1* locus is located approximately 80 kb downstream of the P_C_ allele and was identified by reverse transcription quantitative PCR (RT-qPCR) to be overexpressed in the horn bud region of 90-day polled (P_C_p) fetuses as compared to the horn bud region of wild type (pp) horned fetuses^3^.

Several studies have demonstrated lincRNAs can be cis-acting elements regulating neighboring genes, often located within a few kb of the lincRNA sequence^14–16^. OLIG1, a transcription factor involved in neural crest differentiation pathways^17^, is located approximately 8 kb away from the *lincRNA#1* locus and is the closest gene with a known function. Wiedemar et al.^18^ found that *OLIG1* is overexpressed in the horn bud region of 150 day old horned fetuses when compared to the horn bud region of polled fetuses, and another study investigated how the evolution and development of pecoran (ruminants, excluding Tragulidae) headgear was likely influenced by OLIG1^19^. The inverse correlation between expression of *lincRNA#1* and *OLIG1* suggests an inhibitory or repressive relationship. Downregulation of *OLIG1* expression by upregulation of *lincRNA#1* might be associated with the absence of horn bud development in polled cattle.

In this study, we directly tested whether *lincRNA#1* plays a role in the polled phenotype and investigated a possible regulatory role with respect to *OLIG1*. Little is known about *lincRNA#1*, particularly the functional region, so we decided to take a complete knockout (KO) approach. Using two guide RNAs (gRNAs) with the CRISPR-Cas9 system to create large deletions of greater than 1 kb in length has been successfully achieved in zebrafish^20^ and mice embryos^21^, but has yet to be attempted in livestock. Here, we deployed a dual-guide approach in microinjected zygotes to delete an approximately 3.7 kb region in the bovine genome containing the *lincRNA#1* sequence, but no other known transcripts or proteins, thereby completely knocking out *lincRNA#1*. Presumptive *lincRNA#1* KO bovine embryos were transferred to synchronized recipient cows, and the resulting fetuses were harvested at 90 days of gestion. Fetuses were analyzed to determine if knocking out *lincRNA#1* in a heterozygous (P_C_p) background would result in a horned phenotype, and the expression of both *lincRNA#1* and *OLIG1* expression were assayed using RT-qPCR to investigate their relative expression levels.

## Results

### Bioinformatic analysis

Our analysis indicates that part of the lincRNA#1 sequences in *Bos taurus* are very slightly conserved across 91 vertebrate mammals, but no conserved elements were identified. We have identified 5 other ncRNA sequences (predicted or novel) that have homology to lncRNA#1 (1527 bp), including from bison (LOC105004626; 1261 bp), wild yak (LOC106700733; 1022 bp), goat (ENSCHIG00010001229; 3593 bp) mouse (ENSMUSG00000102958; 660 bp), and dog (ENSCAFG00845026388; 660 bp). Using ClustalW2, the cow lincRNA#1 sequence has 98% similarity to wild yak, 96% similarity to bison, 87% similarity to goat, 85% similarity to mouse and 64% similarity to dog.

### Guide-RNA testing and *lincRNA#1* knockout testing in embryos

**Figure 1:**
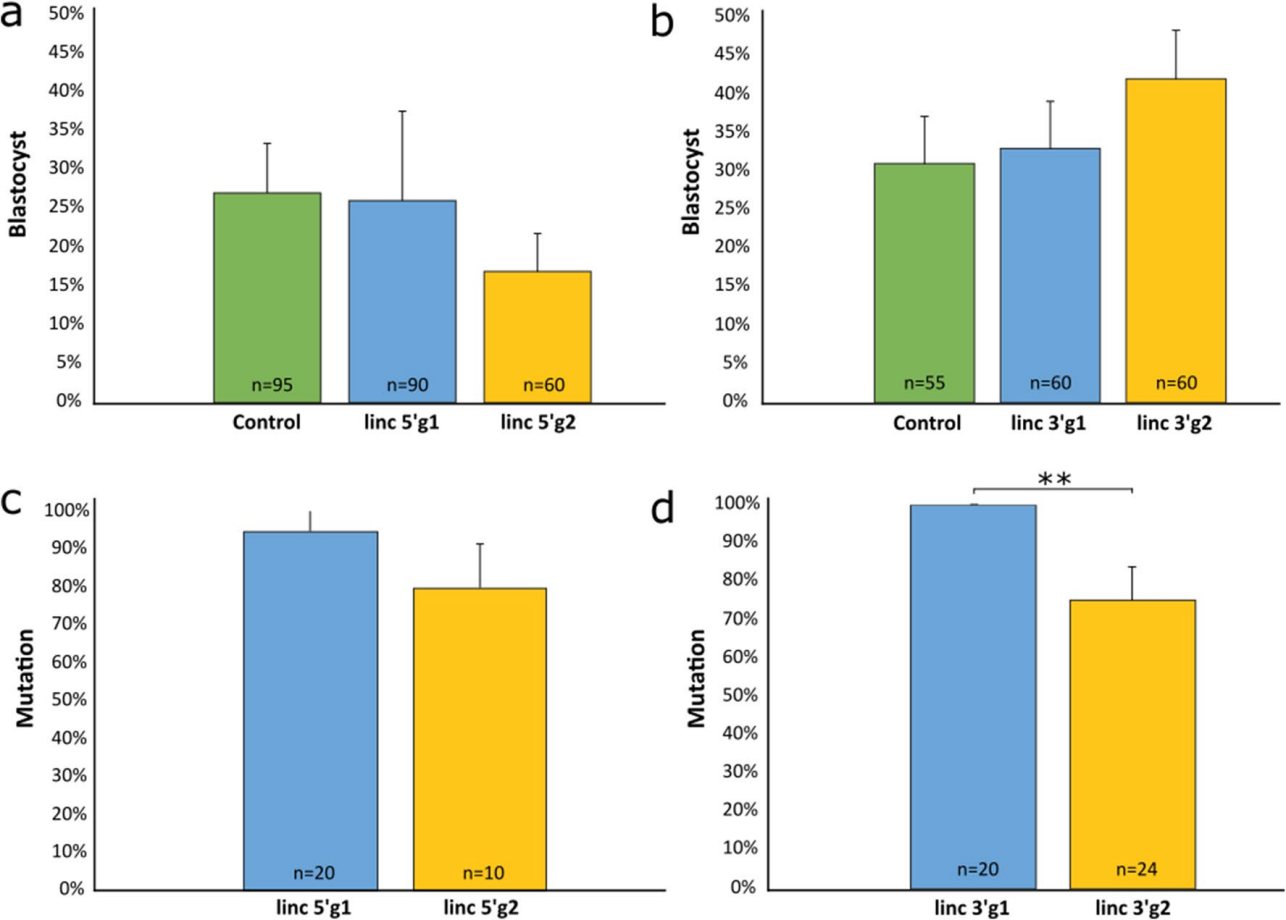
Comparison of uninjected and microinjected zygote development rates and mutation efficiencies. Zygotes were microinjected with gRNA/Cas9 ribonucleoproteins targeting the 5′ and 3′ regions surrounding *lincRNA#1* at 6 h post insemination. Blastocyst development rate of uninjected control (green) and microinjected embryos when targeting the (**a**) 5′ and (**b**) 3′ regions surrounding *lincRNA#1*. Percentage of Cas9-induced mutations in blastocysts when injected with ribonucleoproteins targeting the (**c**) 5′ and (**d**) 3′ regions surrounding *lincRNA#1*. Error bars = SEM. **P < 0.01.

Generating a *lincRNA#1* KO bovine fetus began by designing gRNAs targeting the 5′ and 3′ regions flanking *lincRNA#1* on bovine chromosome 1 (Supplementary Table S1). Using our previously described protocol^22^, we identified two possible gRNAs targeting the 5′ region (linc 5′g1 and linc 5′g2) and two gRNAs targeting the 3′ region (linc 3′g1 and linc 3′g2). The gRNAs were incubated with Cas9 protein to form a ribonucleoprotein (RNP) complex and independently microinjected into zygotes 6 h post insemination (hpi), following a previously established protocol^22^. Uninjected embryos were cultured as a developmental control. For gRNAs targeting the 5′ region, there were no differences in development to the blastocyst stage between uninjected controls (27%), linc 5′g1 (26%), linc 5′g2 (17%), or between the two gRNAs (Fig. 1a; Supplementary Table S2). There were also no differences in mutation rates between linc 5′g1 (95%) and linc 5′g2 (80%) (Fig. 1b, Supplementary Table S2), with a mutation defined as the end product being different than the starting, wild type genome. When testing gRNAs targeting the 3′ region, there was again no difference in blastocyst development between the non-injected control group (31%), linc 3′g1 (33%), linc 3′g2 (42%) groups or between the two test gRNAs (Fig. 1c; Supplementary Table S2). However, there was a difference in mutation rate between linc 3′g1 (100%) and linc 3′g2 (75%; *P* = 0.005) (Fig. 1d, Supplementary Table S2). Due to the higher mutation rate, linc 3′g1 was selected for further analysis.

**Figure 2:**
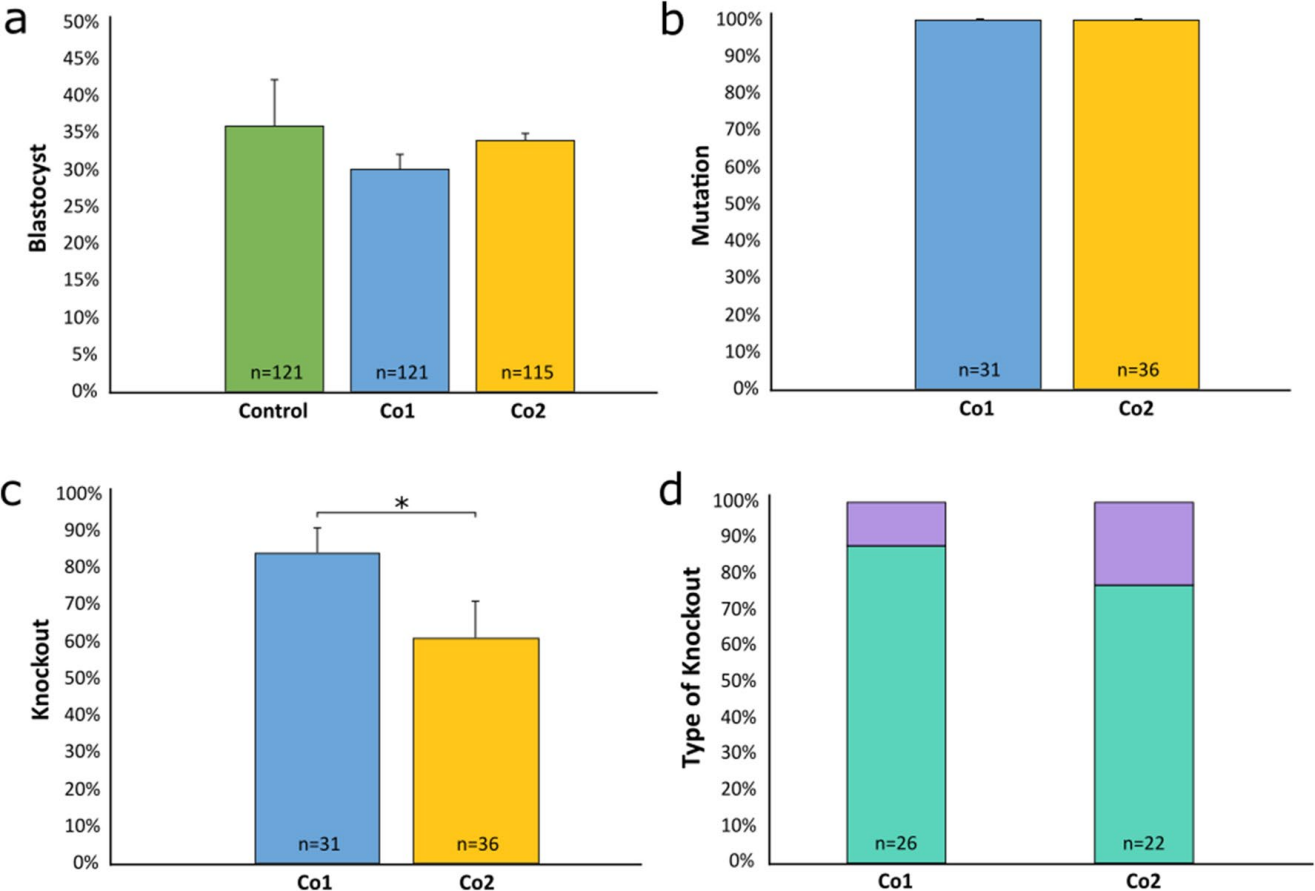
Comparison of uninjected and microinjected zygote development rates and mutation and knockout efficiencies. Zygotes were microinjected with Cas9 protein and either linc 5′g1 and linc 3′g1 (Co1), or linc 5′g2 and linc 3′g1 (Co2), at 6 h post insemination. (**a**) Percentage of uninjected control (green) and microinjected zygotes that reached the blastocyst stage of development. (**b**) Mutation rates and (**c**) knockout rates in embryos injected with gRNA/Cas9 ribonucleoproteins for Co1 (blue) and Co2 (yellow) injection groups. Blastocysts were classified as mutated if a mutation occurred in at least one target site. (**d**) Type of *lincRNA#1* knockout (%) in injected embryos. Bi = biallelic (aqua); Mosaic (purple). Error bars = SEM. *P < 0.05.

To determine the best gRNA combination to use to achieve the *lincRNA#1* KO, two co-injection groups were tested, with co-injection group 1 (Co1) consisting of linc 3′g1 and linc 5′g1 RNP complexes and co-injection group 2 (Co2) consisting of linc 3′g1 and linc 5′g2 RNP complexes. There were no differences in blastocyst development rates between the non-injected controls (36%) and either Co1 (30%) or Co2 (34%) or between the two co-injection groups (Fig. 2a; Supplementary Table S3). There was also no difference in the mutation rate between Co1 or Co2 (both 100%), when a blastocyst was considered positive if a mutation was found in at least one target site (Fig. 2b; Supplementary Table S3). However, the KO rate for Co1 (84%) was 38% higher than the KO rate for Co2 (61%; P = 0.045) (Fig. 2c, Supplementary Table S3). There was an 88% biallelic KO rate and a 12% mosaicism rate in Co1 injected embryos, whereas the biallelic and mosaic KO rates in Co2 injected embryos were 77% and 23%, respectively (Fig. 2d; Supplementary Table S3). No differences were detected between Co1 and Co2 biallelic and mosaic KO rates, however based on the difference in overall KO rates, Co1 was selected as the best combination for producing KO embryos to transfer into synchronized recipient cows.

### Embryo transfers

Once the deletion of *lincRNA#1* was optimized in vitro, recipient heifers were synchronized for embryo transfers. For the first trial (ET1), 15 presumptive edited blastocysts heterozygous (P_C_p) at the *POLLED* locus, and nine presumptive edited blastocysts homozygous (pp) at the *POLLED* locus, which would be expected to be horned (negative controls), were non-surgically transferred into eight synchronized recipients (three blastocysts per recipient) (Table 1). At day 30 of gestation, three out of the eight recipient cows were pregnant based on pregnancy-associated glycoprotein (PAG) testing, one from the heterozygous (P_C_p) embryo transfers, and two from the homozygous (pp) embryo transfers. At day 35 of gestation, transrectal ultrasounds confirmed pregnancies and fetal counts. Two fetuses were detected in the recipient that was carrying the heterozygous (P_C_p) fetuses and five were detected in the recipients carrying the homozygous (pp) fetuses, one carrying twins and the other triplets. Recheck examinations at 75 days of gestation via transrectal ultrasound found only the recipients carrying the five homozygous (pp) fetuses remained pregnant, though none of the fetuses were viable. It was estimated that fetal demise had occurred at roughly 45–50 days of gestation based on fetal measurements. The fetal remnants from these homozygous (pp) fetal demises were recovered the following week for analysis.

**Table 1:** Embryo transfer (ET) results from zygotes injected 6 h post insemination with Cas9 protein and gRNAs linc 5′g1 and linc 3′g1.

ET	Genotype at the *POLLED* loci	Blastocysts transferred	Recipients	35 days of gestation		75 days of gestation		Viable fetuses (%)	Fetuses harvested (%)
				Pregnant (%)	Fetuses detected (%)	Pregnant (%)	Fetuses detected (%)		
1	pp	9	3	2 (67)	5 (56)	2 (67)	5 (56)	0 (0)	5 (56)
	P_C_P	15	5	1 (20)	2 (13)	0 (0)	0 (0)	–	–
2	P_C_P	15	5	4 (80)	5 (33)	4 (80)	5 (33)	5 (33)	5 (33)
Total	pp	9	3	2 (67)	5 (56)	2 (67)	5 (56)	0 (0)	5 (56)
	P_C_P	30	10	5 (50)	7 (23)	4 (40)	5 (17)	5 (17)	5 (17)

Three blastocysts were transferred per recipient. Confirmation of pregnancies was performed on day 35 of gestation and fetal viability at 75 days of gestation. Nonviable fetuses were harvested immediately, and viable fetuses were harvested at 90 days of gestation.

Due to these fetal losses, a second embryo transfer (ET2) was performed, however because of the limited availability of recipients, only blastocysts with the P_C_p genotype at the *POLLED* locus were transferred. These were expected to be polled in the presence of *lincRNA#1* and horned in its absence. A total of 15 heterozygous P_C_p day-7 presumptive *lincRNA#1* KO blastocysts, obtained from two microinjection groups of 50–60 embryos each, were transferred into five synchronized recipients (three blastocysts per recipient) (Table 1). Blastocysts that were not transferred in ET2 were analyzed to establish an editing profile for the transferred embryos. Twelve blastocysts were analyzed, and ten blastocysts had *lincRNA#1* KOs (83%), while the remaining two embryos had mutations only at the linc 3′g1 target site (Supplementary Fig. S1). Of the ten KO embryos, seven were biallelic KOs (70%) and three were mosaic (30%).

At day 29 of gestation, all five recipients were pregnant based on PAG testing. At 35 days of gestation, transrectal ultrasounds confirmed four of the five recipients remained pregnant, with three recipients carrying singletons and one carrying twins. Given the high rate of pregnancy losses from ET1, we performed weekly ultrasounds to monitor fetal development and viability until the five fetuses were harvested at 90 days of gestation.

Overall, nine genetically *HORNED* (pp) edited embryos were transferred into three recipients and 30 genetically *POLLED* (P_C_p) presumptive edited embryos were transferred into 10 recipients (Table 1). At 75 days of gestation for ET1, ultrasounds revealed that fetal demise had occurred, resulting in an overall pregnancy and fetal viability rate of 0%. The fetal remains of the five genetically *HORNED* (pp) fetuses were recovered for analysis. For ET2, four of the ten recipients were pregnant at 75 days of gestation with five viable fetuses (40% pregnancy rate; 17% fetal viability rate), and a total of five genetically *POLLED* (P_C_p) fetuses were harvested at 90 days of gestation.

### Phenotypic and genotypic analysis of fetuses

**Figure 3:**
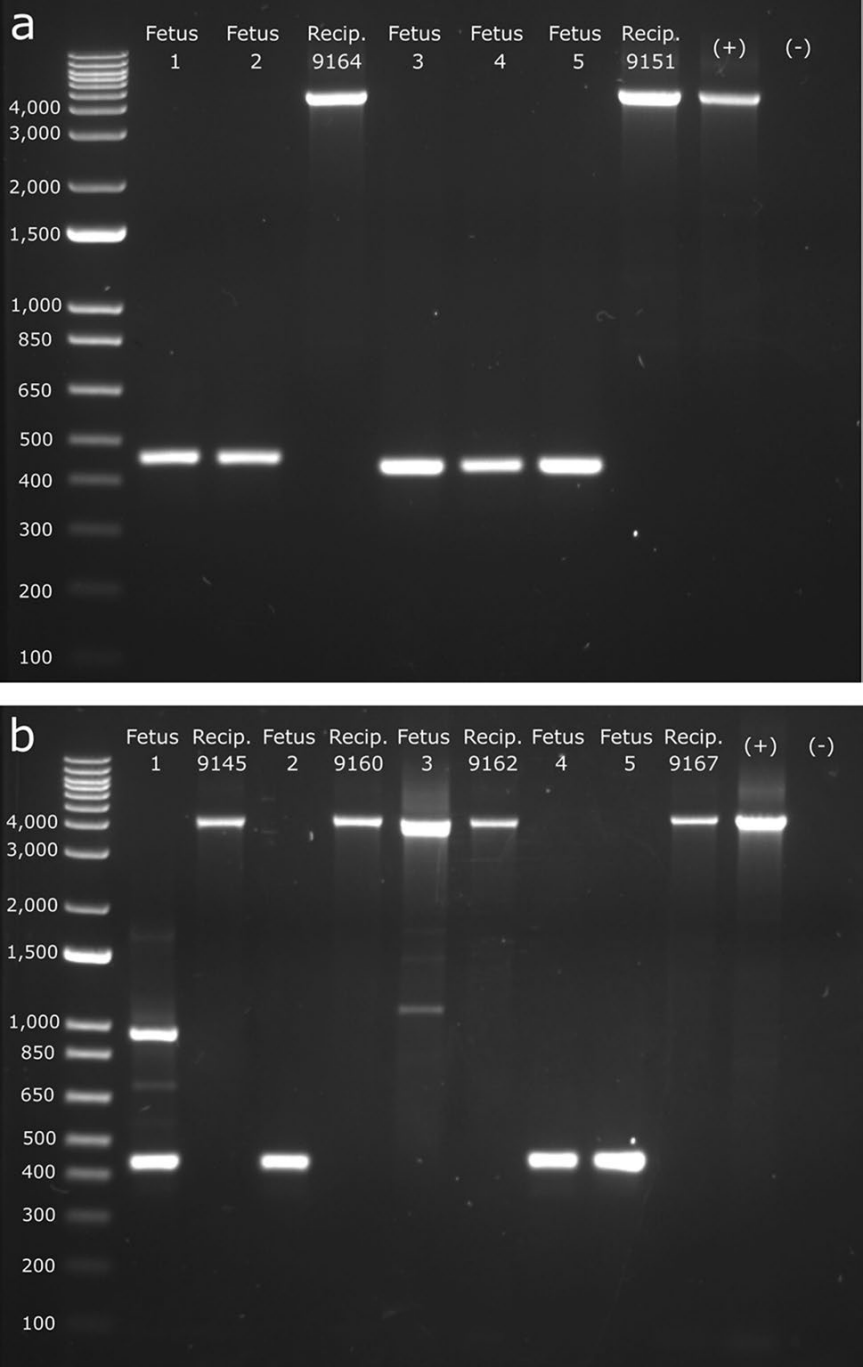
Fetal genotypic analysis from embryo transfers (ETs) and their corresponding recipients (recip.). Gels visualizing the genotypes of fetuses from (**a**) ET1 and (**b**) ET2. DNA was extracted from fetal and recipient tissue and PCR amplified. Gel electrophoresis was done to visualize the *lincRNA#1* targeted deletion. *lincRNA#1* amplicon is 4189 bp and expected knockout size is 456 bp. Recipient DNA follows the respective fetuses they carried.

The fetal remnants from the five ET1 genetically *HORNED* (pp) fetuses were harvested at 83 days of gestation. Two of the fetuses detected in one recipient were almost fully resorbed, and it was estimated they were lost between 35 and 45 days of gestation (Table 2). Three fetuses from the second recipient were not as far along in the resorption process. The basic anatomy was still present, but they were severely degraded. Based on crown-rump length (approximately 2.5–3 cm), it was estimated that fetal demise occurred between 45 and 50 days of gestation. Although the horned phenotype could not be determined due to the age and state of the fetuses, PCR and Sanger sequencing analysis revealed that all five fetuses had biallelic *lincRNA#1* KOs, with fetuses 1 and 2 being compound heterozygous KOs (fetus 1: 3733 bp and 3736 bp deletions; fetus 2: 3,736 bp deletion and a 3740 bp deletion–7 bp insertion) and fetuses 3, 4 and 5 being homozygous biallelic KOs (3755 bp deletions; Fig. 3a; Table 2).

**Table 2:** Fetal genotypes at the target *lincRNA#1* locus from embryo transfers (ETs) of zygotes injected 6 h post insemination with Cas9 protein and gRNAs linc 5′g1 and linc 3′g1.

ET	Days of gestation	Total fetuses	Total mutation (%)	Total knockout (%)	Subset of knockout fetuses		
					Non-mosaic		Mosaic (%)
					Mono (%)	Bi (%)	
1	35–50	5	5 (100)	5 (100)	0 (0)	5 (100)	0 (0)
2	90	5	5 (100)	5 (100)	0 (0)	3 (60)	2 (40)
Total	–	10	10 (100)	10 (100)	0 (0)	8 (80)	2 (20)

A fetus was considered mutated if a mutation was found at one or both gRNA target sites. Knockout rates are further classified in subsets of monoallelic (mono), biallelic (bi) and mosaic. Regarding ET1 where fetal demise occurred, days of gestation refers to how far along the fetuses were in development before they expired, not the days in which they were harvested.

**Figure 4:**
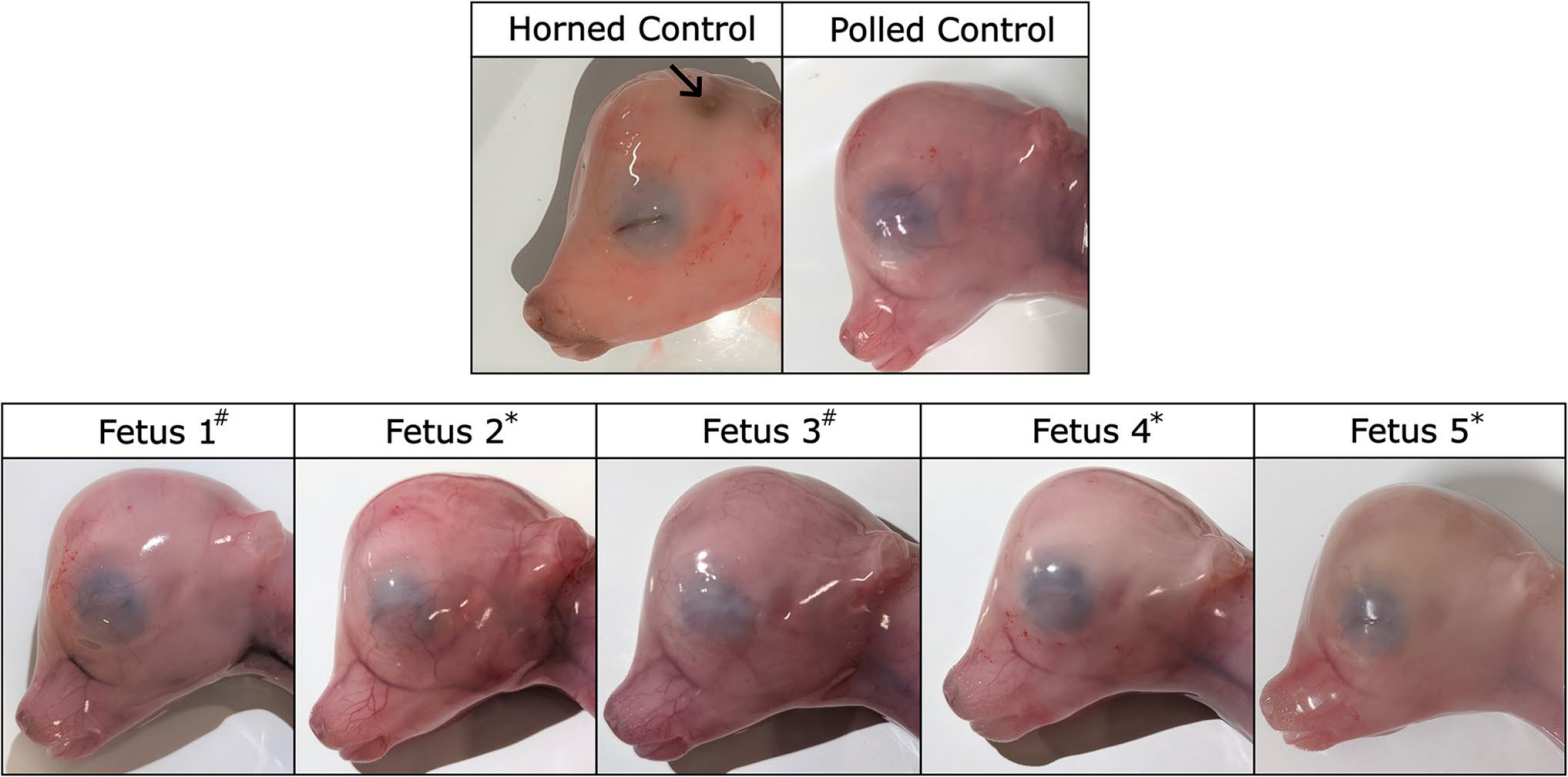
Phenotypic analysis of horn bud development in edited P_C_p fetuses and age-matched unedited horned (pp) and polled (P_C_p) controls. Fetuses were harvested at 90 days of gestation. Black arrow indicates cranial indent indicative of horn bud development. Edited fetuses were #mosaic or *biallelic *lincRNA#1* knockout.

Five genetically *POLLED* (P_C_p) fetuses from ET2 were harvested at 90 days of gestation (Table 2). All five fetuses displayed a polled phenotype (Fig. 4). PCR and Sanger sequencing analysis revealed that fetuses 2, 4 and 5 were homozygous biallelic KO fetuses (fetus 2: 3751 bp deletions; fetuses 4 and 5: 3733 bp deletions; Fig. 3b; Table 2). Fetus 1 was a mosaic KO, and fetus 3 was also a mosaic, with mutations identified at the linc 3′g1 target site and only low KO levels detected. In total, ten (100%) of the edited fetuses contained a *lincRNA#1* KO, however only eight of these (80%) were biallelic KOs. Two (20%) were mosaic with some sequences having an incomplete deletion of the *lincRNA#1* locus (Fig. 3b). Of the sequences that were not the full 3.7 kb *lincRNA#1* KO, none contained an unmutated (wild type) sequence.

### Histological analysis of fetuses

**Figure 5:**
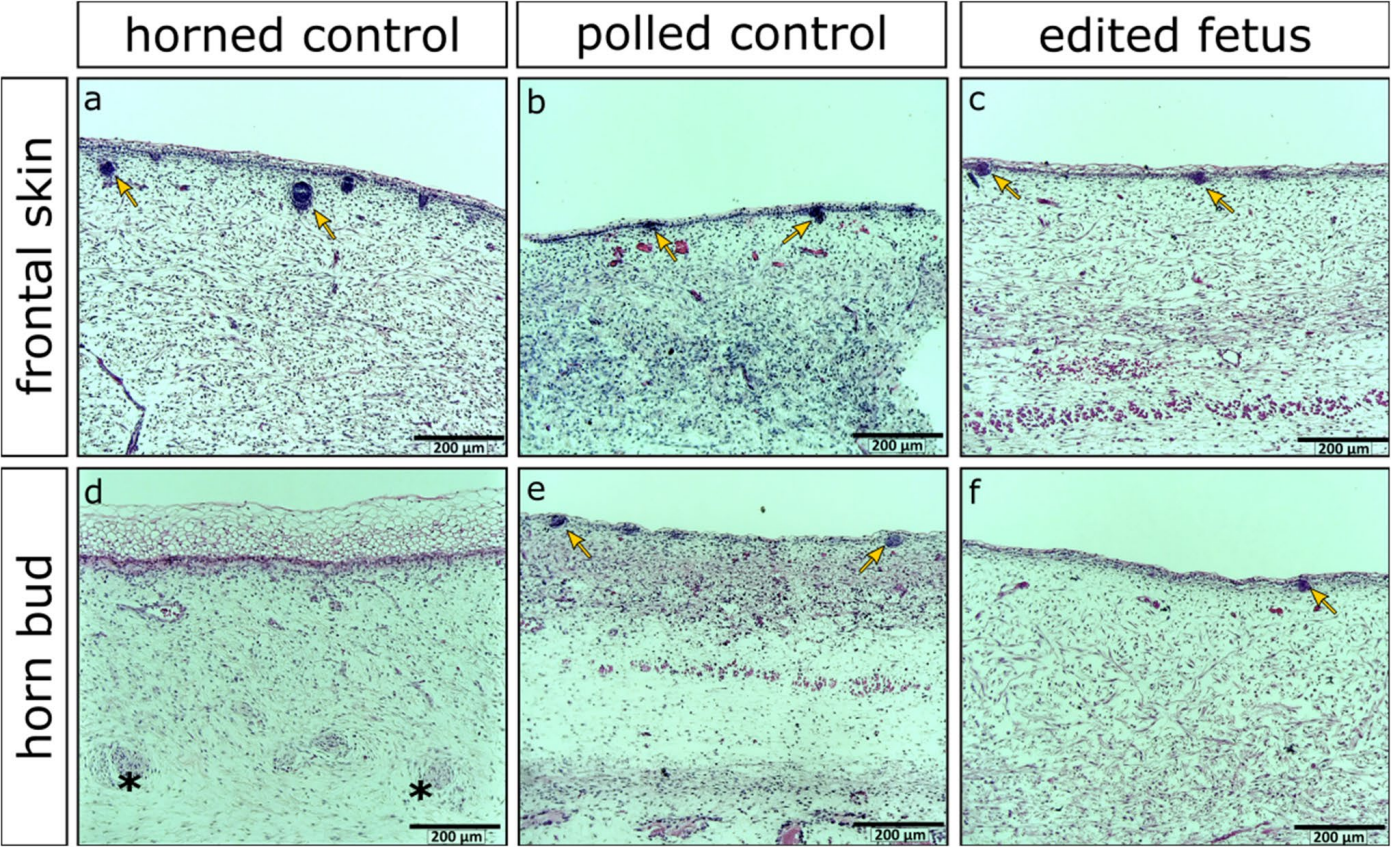
Histological analysis of a representative fetus harvested from embryo transfer (ET) 2 alongside horned and polled controls. (**a**–**c**) Fontal skin and (**d**–**f**) horn bud region of age matched horned and polled control fetuses alongside fetus 2, a representative biallelic *lincRNA#1* knockout fetus from ET2, at 90 days of gestation. Multiple layers of vacuolated keratinocytes and nerve bundles (black stars) can be seen in the horn bud region of the horned control fetus, and hair follicles can be seen in the frontal skin of all fetuses as well as the horn bud regions of the polled control and the knockout fetus (yellow arrows).

Although histological analysis could not be performed on fetuses from ET1, histological analysis of fetuses from ET2 showed the lack of fetal horn bud development consistent with 90-day polled control fetuses (Fig. 5; Supplementary Figs. S2, S3). There was no substantial layering of vacuolated keratinocytes or nerve bundles present in the horn bud region of edited fetuses (Fig. 5f; Supplementary Fig. S2), unlike the horn bud region of horned control fetuses (Fig. 5d). The horn bud regions of edited fetuses were histologically identical to the frontal skin of all fetuses (Fig. 5a–c), with few layers of vacuolated keratinocytes, no nerve bundles, and hair follicles throughout, and were histologically comparable to the horn bud region of the polled control (Fig. 5e; Supplementary Figs. S2, S3).

### Reverse transcription quantitative PCR (RT-qPCR) analysis of fetuses

**Figure 6:**
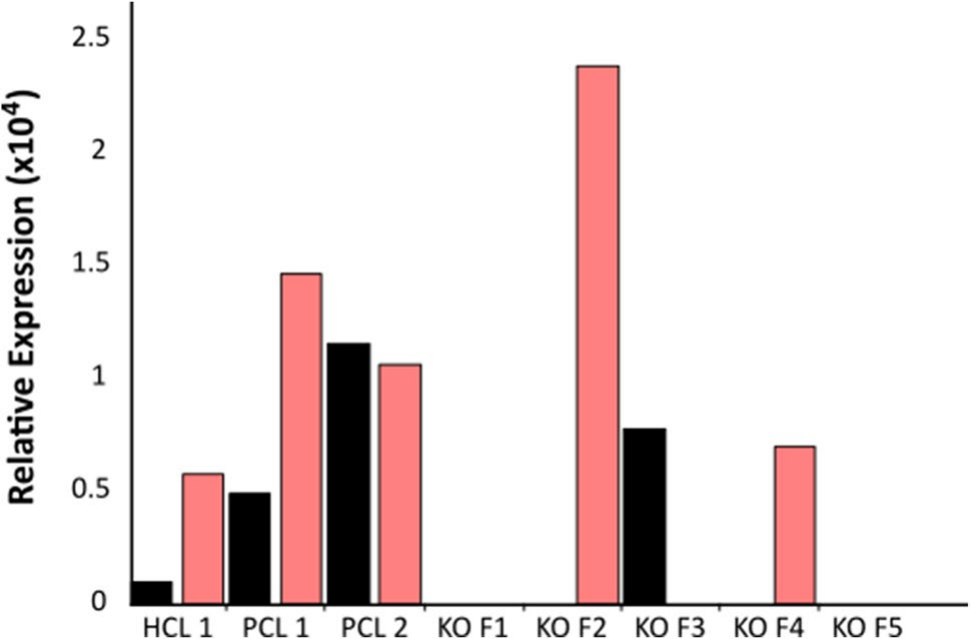
Relative expression of *lincRNA#1* and *OLIG1* in the horn bud region of *lincRNA#1* knockout fetuses (KO F1-5), and one horned (HCL1) and two polled (PCL1 & PCL2) age matched controls. *lincRNA#1* (black) and *OLIG1* (salmon) were normalized relative to three reference genes (*GAPDH*, *RPLP0* and *HPRT1*). *lincRNA#1* KO genotypes are as follows: mosaic KO (F1), biallelic KO (F2), mosaic KO (F3), biallelic KO (F4) and biallelic KO (F5).

RT-qPCR analysis was performed on tissue collected from the horn bud region of edited fetuses from ET2 as well as one unedited horned (pp), and two unedited polled (P_C_p) age-matched controls. We found *lincRNA#1* to have an elevated expression in the horn bud region of the polled control fetuses as compared to the horn bud region of the horned control fetus, although expression varied more than twofold between the two polled controls (Fig. 6). When analyzing the genome edited fetuses, *lincRNA#1* expression was only detected in one mosaic fetus (fetus 3), at nearly identical expression levels as in the polled control, confirming that our biallelic KOs did eliminate this transcript. Due to low sample numbers, no significance can be ascribed to differences in *lincRNA#1* expression levels.

To determine if there was a relationship between *lincRNA#1* and *OLIG1* expression levels, RT-qPCR analysis was also performed on transcripts from both loci. *OLIG1* expression levels were highly variable (Fig. 6). No differences in OLIG1 expression were observed among the different experimental groups, although sample sizes were small. Expression of *OLIG1* tended to be higher in the 90-day polled control fetuses as compared to the horned control, in contrast to the previous study^18^ with 150-day fetuses, however, the highest level of *OLIG1* expression was observed in one of the biallelic *lincRNA#1* KO fetuses (fetus 2). *OLIG1* expression was not detectable in fetus 1 (mosaic) or fetus 5 (biallelic KO), but fetus 4 (biallelic KO) showed similar expression levels to horned controls. The fact that *OLIG1* expression was variably present and absent in *lincRNA#1* biallelic KO fetuses, led us to conclude that knocking out *lincRNA#1* did not have an obvious direct effect on *OLIG1* expression in the horn bud of 90-day old bovine fetuses. It should be noted that expression levels for both *lincRNA#1* and *OLIG1* were extremely low, and detection of both proved challenging in the horn bud region of 90-day old fetuses.

## Discussion

Allais-Bonnet et al. first identified that *lincRNA#1*^3^ was more highly expressed in the horn bud region of polled compared to horned bovine fetuses. LincRNAs may function as cis-acting elements to regulate neighboring genes^14–16^ and *OLIG1*, a gene that codes for a transcription factor involved in neural crest differentiation pathways^17^ that potentially influences horn development^19^, is the closest neighboring gene at approximately 8 kb from the *lincRNA#1* locus. Given previous findings that *OLIG1* is overexpressed in the horn bud region of horned fetuses when compared to the horn bud region of polled fetuses^18^, we hypothesized that the P_C_ allele results in the overexpression of *lincRNA#1* which downregulates the expression of *OLIG1,* thereby inhibiting horn development and resulting in a polled phenotype.

A CRISPR-Cas9 dual-guide approach was deployed in bovine zygotes to achieve large fragment (approximately 3.7 kb) deletion, homozygous KO fetuses, and in this study specifically, KO of *lincRNA#1*. Although we were able to achieve high KO rates, the absence of the *lincRNA#1* transcript, and elimination of any downstream effects of *lincRNA#1* expression, did not result in phenotypically horned fetuses. All genetically *POLLED* (P_C_p) *lincRNA#1* KO fetuses still presented a polled phenotype, suggesting that *lincRNA#1* does not play a role in horn bud suppression and is not responsible for the polled phenotype.

Histological analysis of the genetically *POLLED* (P_C_p) *lincRNA#1* KO fetuses revealed similar morphology to control P_C_p *POLLED* fetuses. At 90 days of gestation, the horn bud and frontal skin regions of polled control fetuses were histologically identical, with abundant hair follicles, few layers of vacuolated keratinocytes, and no nerve bundles present, corresponding to other fetal histological studies^1,3^. This same morphology was seen in our *lincRNA#1* edited fetuses. These histology results support the gross phenotypic observations, indicating that even though our genetically *POLLED* (P_C_p) fetuses had the *lincRNA#1* deletion, they nonetheless presented with a polled phenotype.

There is a small possibility that our polled edited P_C_p fetuses could have developed scurs, horn-like structures that are loosely affixed to the skull^23^. Scurs only occur in heterozygous Pp polled animals regardless of the causal *POLLED* mutation^18^, although the Celtic (P_C_) mutation is more permissive for scur development than the Friesian (P_F_)^23^. The genetic basis for these scurs map to a choice of multiple loci located on BTA19, BTA2, BTA9, BTA10, BTA5, BTA12, BTA 16, BTA18 and BTA23, and the causal mutation(s) are currently unknown^23,24^. The exception to this is the *TWIST1* mutation on BTA4 in Charolais cattle that produces the type 2 scurs phenotype, which is completely independent of a *POLLED* genotype on BTA1^25^. Given the Pp genotype can result in either a true polled or scurred animal depending on several factors such as age, sex and the *POLLED* allele, it indicates that the *POLLED* allele does not have complete dominance regarding the polled phenotype^23^. Since the oocytes used in this study were obtained from a slaughterhouse that culled mostly horned cattle, we were limited to producing heterozygous P_C_p polled fetuses. Three of the five healthy P_C_p fetuses were female which have a significantly lower rate of scur development than males^23^. We would also note that our P_C_p fetuses had similar histological morphology in the horn bud region to the P_C_p fetuses created from polled animals with well-documented pedigrees (having information on at least five generations) that displayed clean polled phenotypes with no scur development^3^. While the likelihood of our fetuses developing scurs is very low, it is still worth noting, given the absence of histological data on the fetal horn bud region in P_C_p animals that do go on to develop scurs.

Analyzing expression levels of *lincRNA#1* and *OLIG1* in the horn bud region of edited and control 90-day bovine fetuses proved challenging. Although we were ultimately able to detect both *lincRNA#1* and *OLIG1* transcripts, the assay required five times the amount of cDNA per qPCR reaction to detect expression in 90-day old bovine fetuses as compared to the study conducted by Allais-Bonnet et al.^3^ (100 ng vs 20 ng, respectively). Wiedemar et al. attempted to analyze *lincRNA#1* and *OLIG1* expression levels across a range of fetal ages, but they were unable to detect these transcripts by qPCR using ~ 16 ng^18^ of cDNA in early stage fetuses, although they were able to detect *OLIG1* expression in 150–158-day old fetuses. It is common for lincRNAs to be minimally expressed, with as little as a few molecules per cell, thus making them extremely difficult to detect^26^. It is possible that cattle breed may also affect *lincRNA#1* expression. Allais-Bonnet et al. used known breeds of cattle (Charolais and Holstein × Normande crossbred cull cows)^3^, whereas we obtained fetuses of unknown breed from a local slaughterhouse, similar to Wiedemar et al.^18^. There was evident (> twofold difference) expression variation with our two polled control fetuses. Even though *lincRNA#1* was difficult to detect, overall our results were consistent with those seen in the study by Allais-Bonnet et al.^3^. It was interesting to observe *lincRNA#1* expression in the mosaic KO fetus (fetus 3), despite the fact that there was no completely wild type sequence of *lincRNA#1* remaining in this fetus. This suggests that whatever mutation was introduced in the sequence did not prevent the expression of *lincRNA#1* and highlights the importance of generating complete KOs of functionally unknown regions/genes to unambiguously study their function.

*OLIG1* also proved difficult to detect and was extremely variable in expression between control and edited fetuses, possibly as a result of its very low concentration in our cDNA preparations. Across the biallelic *lincRNA#1* edited fetuses, *OLIG1* expression varied from non-detectable (fetus 2), to low (fetus 4), to high (fetus 5). Wiedemar et al. also had difficulty detecting *OLIG1* in early developing fetuses, but detection was possible by 150–158 days of gestation^18^. Further studies need to be performed to analyze *OLIG1* expression in *lincRNA#1* KO fetuses at later gestational stages to determine if *lincRNA#1* has any regulatory role over *OLIG1* during gestation.

High rates of embryonic loss and death were observed in the first embryo transfer performed in this study, with all the fetuses recovered being nonviable. Abortion diagnostic panels were performed on the recipients to rule out infectious causes for the fetal losses, but no diagnosis was reached. Because the function of *lincRNA#1* is unknown, we speculated that it could be a lethal KO, however several studies have shown that long non-coding RNAs are dispensable, and their absence does not result in the organism’s death^15,17,27^. A second embryo transfer was performed with the same recipient pool and veterinarian, and a much higher rate of success was achieved. Since all the fetuses harvested from the second embryo transfer were viable at 90-days and three were complete biallelic *lincRNA#1* KOs, it was determined to be a non-lethal KO, leaving the exact cause of the fetal demises observed in ET1 undiagnosed.

Mosaicism is a common problem observed when using genome editing reagents in developing embryos. Our previous work reported an inverse correlation between mosaicism rate and timing of injections hours post insemination (hpi), with the lowest mosaicism rates being seen in embryos edited 6 hpi^28^. Interestingly, we saw this same trend in the work presented here. Based on the blastocyst developmental rates in our preliminary studies, it was determined that around 100–120 embryos would be needed to obtain sufficient blastocysts for embryo transfers. To ensure editing reagents were at their highest editing efficiency and to limit the time embryos were exposed to suboptimal conditions outside of the incubator, two groups of 50–60 zygotes were injected separately using newly prepared editing reagents per injection group. This means that one group (group 1) was injected 6 hpi and the second group (group 2) was injected around 6.5–7 hpi. Any remaining blastocysts not transferred to recipients were analyzed to obtain an editing profile of those embryos that were transferred. Although all the embryos from ET1 were transferred, there were 12 non-transferred embryos from ET2 that were analyzed, eight from group 1 and four from group 2. It was interesting to observe that the deletion rate was higher and the mosaicism rate was lower in embryos injected in group 1 as compared to embryos injected group 2 (Supplementary Fig. S1). Furthermore, of the edited fetuses harvested, the two mosaic fetuses were both from group 2 embryos. These results lend further support to the importance of early introduction of genome editing agents into zygotes to promote high editing efficiency and low mosaicism rates. These data also highlight one of the downfalls of microinjection. Microinjection is time consuming, even if done by a skilled individual. A better approach would be to edit all embryos simultaneously in a shorter amount of time using recently developed electroporation techniques^29^. The optimization of electroporation methods to simultaneously introduce editing reagents into hundreds of livestock zygotes may increase the obtainment of genome edited biallelic KO embryos with low mosaicism rates.

Overall, this paper describes how a CRISPR-Cas9 dual-guide approach was used to create a large (> 3 kb) targeted deletion in bovine embryos. Embryo transfers of presumptive *lincRNA#1* KO embryos were performed, and although all of the pregnancies from the first embryo transfer resulted in fetal demise, five phenotypically normal 90-day fetuses were harvested from the second embryo transfer for analysis. Of those five genotypically *POLLED* (P_C_p) fetuses, all carried a deletion containing *lincRNA#1,* and all but one mosaic fetus showed no *lincRNA#1* expression. However, all fetuses presented with a polled phenotype. Based on these results, we established that absence of *lincRNA#1* alone does not result in a horned phenotype, suggesting that *lincRNA#1* expression is not required for horn bud suppression.

## Materials and methods

### Animal care

All experimental protocols and methods including animals were conducted in compliance with the ARRIVE guidelines and in accordance with the University of California. The study was approved by Institutional Animal Care and Use Committee (IACUC) protocol #20746 at the University of California, Davis. Recipient cattle were housed and managed at the University of California, Davis Feedlot.

### Bioinformatic analysis

We examined the conservation track for 91 vertebrate mammals in Ensembl (www.ensembl.org) for the gene *ENSBTAG00000054023* (equivalent to *LOC100848368* from Genbank) as compared to the highly conserved neighboring *OLIG1* gene. The genomic sequence encoding the longest *LOC100848368* transcript (NC_037328.1:2506495-2509757 in ARS-UCD1.2) was compared in Ensembl against the genomes of a wide variety of species (37 total, ranging from Wallaby to Australian saltwater crocodile) using BLAST (https://uswest.ensembl.org/Multi/Tools/Blast) to find ncRNA sequences encoded by genomic sequences with significant homology to *LOC100848368*. The longest transcript of *LOC100848368* was also compared against the nucleotide database in Genbank to find homologous ncRNA sequences using BLAST (https://blast.ncbi.nlm.nih.gov/Blast). We aligned the 6 ncRNA sequences using multiple sequence alignment of RNA in ClustalOmega (https://www.ebi.ac.uk/Tools/msa/clustalo/).

### Control fetal collections

Control horned and polled fetuses were either collected from a local processing plant or as part of separate ongoing departmental experiments. Time of gestation was estimated based on the crown-rump length calculator (University of Wisconsin-Madison) when an exact gestational age was not known. Phenotype was identified, the fetal heads were separated (sagittal) and the horn bud and frontal skin regions were processed for either histological or RT-qPCR analysis.

DNA was isolated from tail tissue samples by means of Qiagen’s DNeasy Blood & Tissue Kit (Valencia, CA), and *POLLED* genotyping was done by polymerase chain reaction (PCR) to determine if they carried either *POLLED* (P_C_ or P_F_) alleles (primer sequences in Supplementary Table S4). 100 ng of DNA was amplified using GoTaq^®^ Green Master Mix (Promega, San Luis Obispo, CA) and 400 nM of each primer on a SimpliAmp Thermal Cycler (Applied Biosystems, Waltham, MA) for 5 min at 95 °C, 35 cycles of 30 s at 95 °C, annealing for 30 s (temperatures are in Supplementary Table S4), and extension at 72 °C (times are in Supplementary Table S4), followed by 10 min at 72 °C. Products were electrophoresed and visualized using 1% Tris-Acetate Ethylenediamine Tetra-Acetic Acid (TAE) agarose gels. PCR products were gel-extracted using a modified version of the “freeze-squeeze” method^30^. Briefly, filter columns were prepared by cutting the ends (approximately 3–4 mm) of p20 filter tips (Mettler-Toledo) and placing them in 1.5 mL tubes. Bands were excised from the gel, placed into the filter columns, incubated at − 80 °C for 5 min and centrifuged at max speed for 3 min. The filter tip containing the agarose was discarded and the filtrate containing the DNA was sent for Sanger sequencing (GENEWIZ, San Francisco, CA).

### Guide RNA design and construction

The online tools sgRNA Scorer 2.0^31,32^ and Cas-OFFinder^33^ were used to design guide RNAs targeting the 5′ and 3′ regions flanking *lincRNA#1* (*LOC100848368*) on chromosome 1 of the UMD3.1.1 bovine reference genome^34^. Guide selection was done with the requirements of no less than three mismatches in the guide sequence for off-target sites in the genome with at least one mismatch in the seed region (8–11 bp upstream of the PAM sequence). The top two guides for each target (Supplementary Table S1) were ordered from Synthego (Menlo Park, CA) with no modifications of the gRNAs. In vitro cleavage assays were done to test cleavage efficiency by incubating 80 ng of PCR amplified target sequence, 100 ng of gRNA, 150 ng of Cas9 protein (PNA Bio, Inc., Newbury Park, CA), in 1× Buffer 3.1 (New England Biolabs, Ipswich, MA) at 37 °C for 1 h. Products were electrophoresed and imaged using a 2% agarose gel.

### Embryo production

Bovine ovaries were obtained from an abattoir and transported to the laboratory in 35–37 °C sterile saline. Collection of cumulus-oocyte complexes (COCs) was done via aspiration of follicles and groups of 50 COCs were matured in 4-well dishes containing 500 μL of maturation media (BO-IVM, IVF Bioscience, Falmouth, United Kingdom). COC maturation was done in a humidified 5% CO_2_ incubator at 38.5 °C for 20–22 h. Oocytes were fertilized in groups of 25 per drop (60 μL) of SOF-IVF^35^ covered with OVOIL (Vitrolife, Sweden). Holstein *HORNED* homozygous pp or Angus *POLLED* homozygous P_C_ semen was used to fertilize homozygous pp oocytes to create embryos for the horned and polled embryo groups by IVF. A concentration of 2 × 10^6^ sperm per mL was used for an incubation period of 6 h at 38.5 °C in a humidified 5% CO_2_ incubator. Light vortexing in SOF-HEPES medium^35^ was done for 5 min to denude presumptive zygotes of cumulus cells. No more than 100 zygotes per well were incubated in 400 μL of culture media (BO-IVC, IVF Bioscience) covered with 300 μL of OVOIL at 38.5 °C in a humidified atmosphere of 5% CO_2_, 5% O_2_ and 90% N_2_ for 7–8 days.

### Guide-RNA testing

To determine gRNA mutation rates, laser-assisted cytoplasmic microinjection^36^ of presumptive zygotes was performed with 6 pL of a mixture of 67 ng/μL of gRNA and 167 ng/μL of Cas9 protein (PNA Bio) incubated at room temperature for 30 min prior to injection. Embryos were incubated for 7–8 days and those that reached the blastocyst stage were lysed in 10 μL of Epicenter DNA extraction buffer (Lucigen, Palo Alto, CA) at 65 °C for 6 min then 98 °C for 2 min. The target regions were amplified by PCR using primers designed with Primer3 (Supplementary Table S4)^37,38^. A nested PCR approach was undertaken with the first round of PCR containing 10 μL GoTaq^®^ Green Master Mix (Promega), 200 nM of each primer and 9.2 μL of DNA in lysis buffer for 5 min at 95 °C, 35 cycles of 30 s at 95 °C, 30 s annealing (temperatures in Supplementary Table S4), and 72 °C extension (times in Supplementary Table S4) at followed by 5 min at 72 °C. The second round of PCR was run on 1 μL of first round PCR reaction using GoTaq^®^ Green Master Mix (Promega) with 200 nM of each primer for 3 min at 95 °C, 35 cycles of 30 s at 95 °C, 30 s annealing (temperatures in Supplementary Table S4), and extension at 72 °C (times in Supplementary Table S4), followed by 5 min at 72 °C. Products were electrophoresed and visualized on a 1% agarose gels, excised and purified using the QIAquick Gel Extraction Kit (Qiagen, Valencia, CA). The DNA was then Sanger sequenced (GENEWIZ), and analyzed using CRISP-ID^39^ and ICE^40^.

Mutation rates for co-injected IVF embryos were determined using the same methods described above utilizing one of the two 5′ gRNAs with the most efficient 3′ gRNA (67 ng/μL of each guide) alongside 167 ng/μL of Cas9 protein (PNA Bio). Blastocysts were collected as previously described and the target region was amplified using a nested PCR approach with primers designed using Primer3 (Supplementary Table S4)^37,38^. The first round of PCR was performed on 9.5 μL of DNA in lysis buffer using LongAmp^®^ Taq Master Mix (New England Biolabs) and 400 nM of each primer for 3 min at 94 °C, 35 cycles of 30 s at 94 °C, 30 s at 63 °C, and 4 min 30 s at 65 °C, followed by 10 min at 65 °C. The second round of PCR was run on 1 μL of first round PCR using GoTaq^®^ Green Master Mix (Promega) and 200 nM of each primer for 3 min at 95 °C, 35 cycles of 30 s at 95 °C, 30 s at 64 °C, and 4 min 15 s at 72 °C, followed by 10 min at 72 °C. PCR products were electrophoresed and visualized on a 1% TAE agarose gel then excised and purified using the modified “freeze-squeeze” method described above. The DNA was Sanger sequenced (GENEWIZ), and analyzed using CRISP-ID^39^ and Mixed Sequences Reader^41^.

### Embryo transfers

Recipient cattle estrus synchronization began 16 days preceding the embryo transfer. On day 0, recipients received an intravaginal progesterone releasing device (1.38 g; Eazi-Breed CIDR; Zoetis) and gonadorelin (100 μg; Factrel; Zoetis). The CIDRs were removed and prostaglandin (25 mg; Lutalyse; Zoetis) was administered on day 7, then a second dose of gonadorelin (100 μg; Factrel; Zoetis) was given on day 9 while recipients were monitored for signs of estrus. On day 9 of synchronization, presumptive zygotes were injected with linc 5′g1 and linc 3′g1 RNP complexes as described above. Embryos were injected in groups of 50–60, and fresh editing reagents were prepared between each group. Recipient synchronization was confirmed on day 15 via detection of a corpus luteum using a transrectal ultrasound (5.0 MHz linear probe; EVO Ibex, I.E. Medical Imaging). Embryo transfers were performed on day 16. A caudal epidural of 100 mg 2% lidocaine (Xylocaine; Fresenius) was administered to recipients prior to embryo transfer. Straws (0.25 cc) were loaded with three blastocysts each and transferred into the uterine horn ipsilateral to the corpus luteum using a non-surgical transcervical technique. Any remaining blastocysts that were not transferred were analyzed via PCR and Sanger sequencing as described previously to get an editing profile of embryos transferred. On day 28 of embryonic development, recipient cow blood was drawn to diagnose pregnancy via PAG detection, and on days 35 and 75, transrectal ultrasonography was performed for confirmation of pregnancy. Recipients were resynchronized for subsequent embryo transfers if they did not become pregnant from prior embryo transfers.

### Phenotypic and genotypic analysis of fetuses

At 90 days of gestation, recipient cattle were slaughtered via penetrating captive bolt and subsequent exsanguination. The reproductive tracts were collected, fetuses were recovered from the uterine horns, and horn bud phenotyping was performed. Fetal liver and tail tissue samples were collected for DNA extraction and recipient muscle tissue was harvested for experimental controls. The frontal skin and horn bud regions were collected for histological and RT-qPCR analysis.

Fetal genotypes were determined via DNA extraction from tissue samples using the DNeasy Blood & Tissue Kit (Qiagen). 80 ng of DNA was PCR amplified using GoTaq^®^ Green Master Mix (Promega) and 400 nM of each primer for 3 min at 95 °C, 40 cycles of 30 s at 95 °C, 30 s at 62 °C, and 4 min at 72 °C, followed by 10 min at 72 °C. PCR products were electrophoresed and visualized on 1% TAE agarose gels. DNA was extracted using the “freeze-squeeze” method previously described. Fetuses were also tested for the P_C_ and P_F_ alleles at the *POLLED* locus utilizing the PCR protocol that was described for control fetal collections.

### Histological analysis of fetuses

Tissue samples from the horn bud and frontal skin were fixed in 4% paraformaldehyde (Thomas Scientific, LLC, Swedesboro, NJ) for 18 h at 4 °C. Samples were washed in phosphate-buffered saline three times on a rocker for 30 min and placed in 70% ethanol. Tissues were dehydrated in a graded ethanol series and cleared with xylene in a vacuum infiltration processor (Sakura Tissue-Tek VIP 5, Torrance, CA). Samples were then embedded in paraffin blocks, cut in 5 µm sections using a Leica RM2255 microtome (Leica Biosystems, Buffalo Grove, IL) and stained with hematoxylin and eosin. Visualization was done with an Echo Revolve microscope (Discover Echo Inc., San Diego, CA).

### Reverse transcription quantitative PCR analysis of fetuses

Horn bud tissue disruption was done under liquid nitrogen using a mortar and pestle, and RNA was extracted using TRIzol (Invitrogen, Waltham, MA) per the manufacturer’s instructions. The RNA (5 µg) was treated using RQ1 RNase-free DNase (Promega, San Luis Obispo, CA), and cDNA was synthesized from 2 µg of RNA using SuperScript II Reverse Transcriptase (RT; Invitrogen, Waltham, MA), random hexamers (Invitrogen, Waltham, MA) and oligo dT (Promega, San Luis Obispo, CA) alongside appropriate positive and negative controls. To confirm successful DNase treatment and cDNA synthesis, 40 cycles of PCR was performed on the cDNA alongside RT negative controls (lacking either RT enzyme or RNA) using *RPLP0* and *HPRT1* primers as described above, and products separated on a 1% TAE gel. A standard curve for quantitative PCR (qPCR) was made using a 7-point fivefold dilution series of either cDNA (for reference genes; 100 ng/reaction) or genomic DNA (for target genes; 100 ng/reaction) and used with every qPCR assay. PowerUp SYBR mix (Applied Biosystems) was used for qPCR of either 20 ng (for reference genes), or 100 ng (for target amplicons) of cDNA, all in triplicate. Primers were either found in the literature^3^ or were designed as described above, spanning exon/exon junctions when possible (Supplementary Table S4). qPCR was performed on the QuantStudio 3 Real-Time PCR System (Applied Biosystems) using the following program: 50 °C for 2 min, 95 °C for 10 min, followed by 40 cycles of 95 °C for 15 s, 60 °C for 1 min, followed by a dissociation curve. Three reference genes (*GAPDH*, *HPRT1* and *RPLP0*) were used, and normalization of target gene expression was done using the formula: ${\text{Y}}_{\text{i}}= \frac{{\text{Q}}_{\text{Ti x }}{\text{dil}}_{\text{T}}}{{\text{dil}}_{\text{R }}\text{x }\sqrt[3]{{\text{Q}}_{\text{R}1\text{i}}\text{ x }{\text{Q}}_{\text{R}2\text{i}}\text{ x }{\text{Q}}_{\text{R}3\text{i}}}}$ where Y_i_ is the normalized target gene expression for the ith fetus, Q_Ti_ and Q_Ri_ are target and reference gene quantities, respectively, and dil_T_ and dil_R_ are the dilution factors used for the cDNA target and reference amplicons^42^.

### Statistical analysis

Comparison between blastocyst development, mutation and KO rates were evaluated using a binomial logistic regression model in R with gRNA modeled as a fixed effect. Differences were considered significant when *P* < 0.05.

## Supplementary Information

Supplementary Information.

## Data Availability

Data produced and evaluated in this study are included in this published article and its corresponding Supplementary Information file.
