# Supplementary material for: LincRNA#1 knockout alone does not affect polled phenotype in cattle heterozygous for the celtic POLLED allele
Source: Sci Rep. 2022 May 10;12:7627. doi: 10.1038/s41598-022-11669-9 (PMC9090918; doi:10.1038/s41598-022-11669-9)
Supplement: Supplementary file 1 — Supplementary Information. [file 41598_2022_11669_MOESM1_ESM.pdf]

Supplementary Data

LincRNA#1 Knockout Alone does not Affect Polled Phenotype in Cattle Heterozygous for the Celtic *POLLED* Allele

Sadie L. Hennig[1], Bret R. McNabb[2], Josephine F. Trott[1], Alison L. Van Eenennaam[1] and James D. Murray[1,2]

[1]Department of Animal Science, University of California – Davis, Davis, CA

[2]Department of Population Health and Reproduction, School of Veterinary Medicine, University of California – Davis, Davis, CA

Corresponding Author: jdmurray@ucdavis.edu

SUPPLEMENTARY DATA

Supplementary Table S1. Guide RNAs targeted the 5’ and 3’ flanking sequence of *lincRNA#1*.

Target Region	Name	Sequence
5’ of *lincRNA#1*	linc 5'g1	TGCGGGCAGATGTCTTGCCG
	linc 5'g2	TCTGAGCTGCTGAAGTGTGC
3’ of *lincRNA#1*	linc 3'g1	GTTGCTTGAACGCTCTGCGA
	linc 3'g2	TCTGCCTAAAATTCGGTTAA

Supplementary Table S2. Rate of blastocyst development and mutations in zygotes after microinjection of gRNA/Cas9 ribonucleoproteins targeting the 5' and 3' regions of *lincRNA#1*. *In vitro* fertilized bovine embryos were microinjected 6 hours post insemination. On day 8, blastocysts were counted, and Sanger sequencing was done to determine mutations. [A,B,a,b]Letters that differ in the same column are significantly different; [A,B]$P < 0.01$; [a,b]$P < 0.05$.

Target Region	gRNA	Total Embryos	Total Blastocysts (%)	Total Analyzed	Total Mutations (%)
5'	Non-Injected Control	95	26 (27)[a]	-	-
	linc 5'g1	90	23 (26)[a]	20	19 (95)[a]
	linc 5'g2	60	10 (17)[a]	10	8 (80)[a]
3'	Non-Injected Control	55	17 (31)[A]	-	-
	linc 3'g1	60	20 (33)[A]	20	20 (100)[A]
	linc 3'g2	60	25 (42)[A]	24	18 (75)[B]

Supplementary Table S3. Blastocyst, mutation, and targeted knockout rates of zygotes following microinjection of Cas9 protein, linc 3'g1 and linc 5'g1 (Co1) or linc 5'g2 (Co2). *In vitro* fertilized bovine embryos were microinjected 6 hours post insemination. On day 8, blastocysts were counted, and Sanger sequencing was done to determine mutations. Blastocysts were categorized as mutated if a mutation occurred in at least one target site. [a,b]Letters that differ in the same column are significantly different ($P < 0.05$).

Time of Injection	Total Embryos	Total Blastocysts (%)	Total Analyzed	Total Mutation (%)	Total Deletion (%)	Subset of Deletion Embryos		
						Non-Mosaic		Mosaic (%)
						Mono (%)	Bi (%)	
Non-Injected Control	121	44 (36)[a]	-	-	-	-	-	-
Co1	121	36 (30)[a]	31	31 (100)[a]	26 (84)[a]	0 (0)[a]	23 (88)[a]	3 (12)[a]
Co2	115	39 (34)[a]	36	36 (100)[a]	22 (61)[b]	0 (0)[a]	17 (77)[a]	5 (23)[a]

Supplementary Table S4. PCR primers used for amplification of *lincRNA#1* target regions and P_C and P_F alleles. Quantitative PCR (qPCR) primers used for quantification of *lincRNA#1* and *OLIG1* transcripts and reference genes. BLs = blastocysts; KO = knockout.

**Target**	**PCR Round**	**Forward**	**Reverse**	**Tm (°C)**	**Extension Time**
linc 5'guides	1st	ACACGACTGAGCAGCTAACT	ACTCTCTGTGACCGCATGAA	60	1 min
	2nd	GACAGGGTGGAAAGACAAGC	GGTCTTTTCCAATGAGCCCC	62	30 s
linc 3' guides	1st	AGGGAGTGCAAGTTGATCCA	TATGGCCAGAATCGCTCACA	60	1 min 30 s
	2nd	TGTCTGACTCCTTGCAACCA	CCTTTGAAACGTTTGGCTCC	60	1 min 15 s
lincRNA#1 KO in BLs	1st	CGTGTGGATACCTCTCAGCT	CTGGCTGTTTTAGTCTGGGC	63	4 min 30 s
	2nd	AGGGAGTGCAAGTTGATCCA	GGTCTTTTCCAATGAGCCCC	64	4 min 15 s
lincRNA#1 KO in fetuses	1st	TGGACACGTGTGGATACCTC	GCCATCCCAGTGGTGAGAAC	62	4 min
P_C allele	1st	GAAGTGTGGCCGGTAGAAAA	TCCGCATGGTTTAGCAGGATTCA	60	1 min
P_F allele	1st	CCATCTTGGGTACAGCGTTT	TGTTCTGTGTGGGTTTGAGG	60	30 s
lincRNA#1 (qPCR)	1st	ACCAGGAGGGGAGAAAGAAA	TTCGGGAGAGGAAGGAGGT	60	30 s
OLIG1 (qPCR)	1st	CATCATCGCGACAAAACATC	AATTCCCAGGTCGATGAGTG	60	30 s
GAPDH (qPCR)	1st	TTCAACGGCACAGTCAAGG	ACATACTCAGCACCAGCATCAC	60	30 s
RPLP0 (qPCR)	1st	TCTCCTTCGGGCTGGTCAT	AGGAAGCGGGAATGCAGAGT	60	30 s
HPRT1 (qPCR)	1st	GAACGGCTGGCTCGA	TCCAACAGGTCGGCAAAGAA	60	30 s

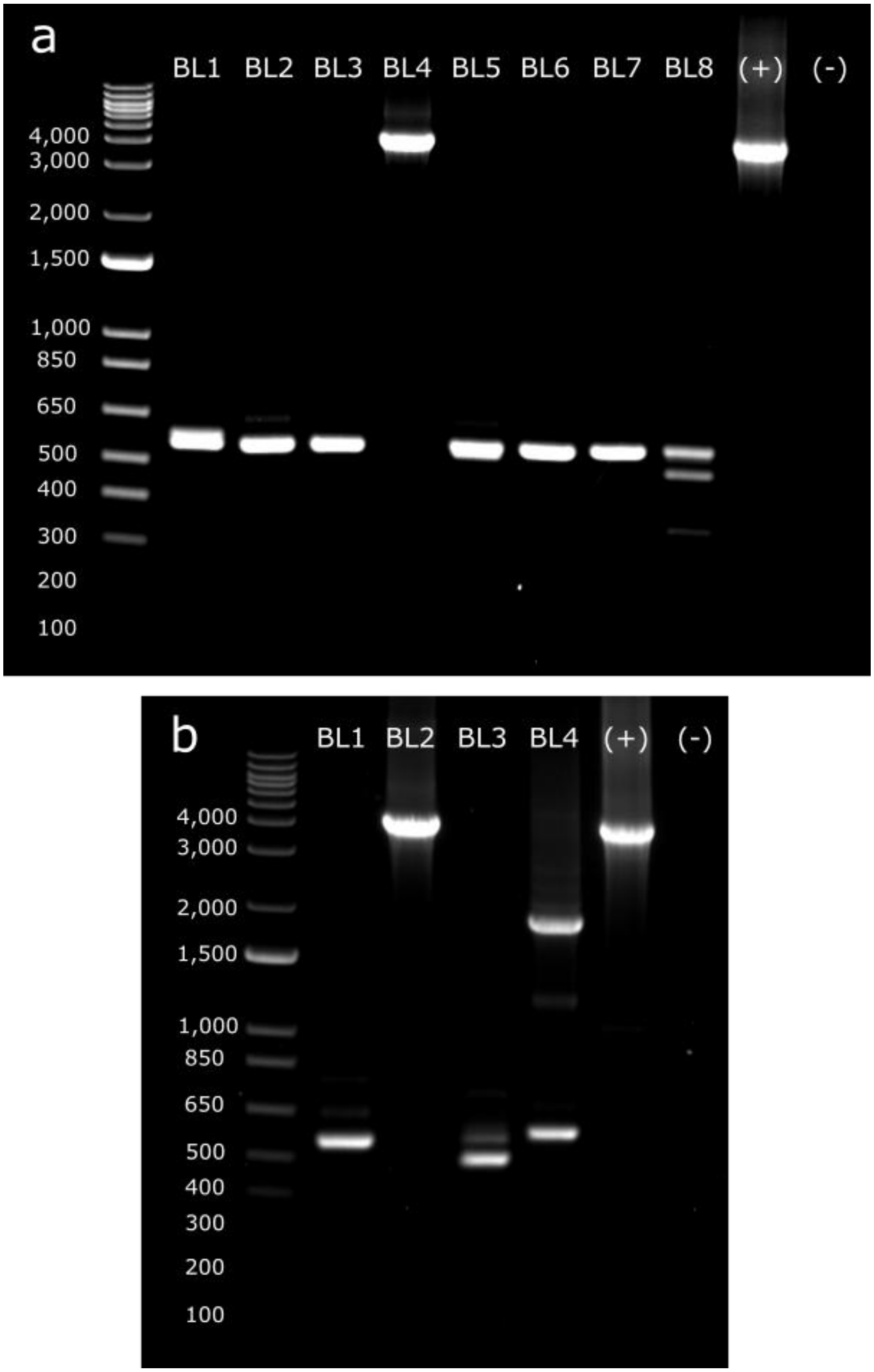

Supplementary Figure S1. Knockout detection at the *lincRNA#1* locus of remaining embryos not transferred for embryo transfer 2. (**a**) Remaining embryos co-injected with Cas9 protein and linc 5'g1 and linc 3'g1 in the first injection group (group 1) and (**b**) second injection group (group 2). Following culture to day-8 blastocysts (BLs), DNA was extracted, PCR amplified and gel electrophoresis was done. Wild type *lincRNA#1* amplicon is 4,287 bp and expected size with deletion is 554 bp.

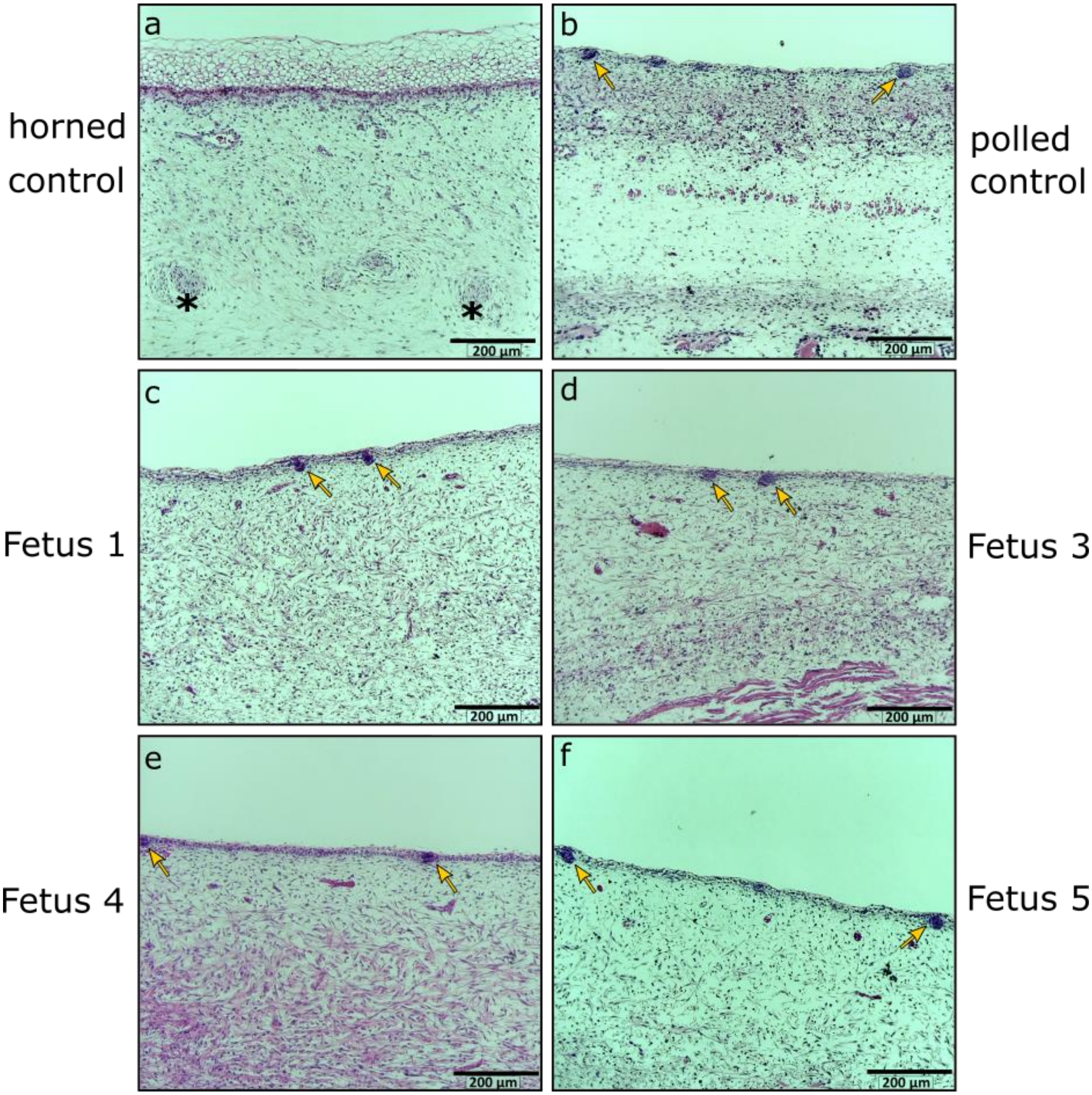

Supplementary Figure S2. Horn bud histological analysis of 90-day fetuses harvested from embryo transfer (ET) 2 alongside horned and polled controls. (**a**) Horn bud of horned and (b) polled age matched control fetuses alongside (c-f) the horn bud regions of *lincRNA#1* knockout (KO) fetuses at 90 days of gestation. Multiple layers of vacuolated keratinocytes and nerve bundles (black stars) can be seen in the horn bud region of the horned control fetus, and hair follicles can be seen in the horn bud regions of the polled control and edited fetus (black arrows). Stained with hematoxylin and eosin. Fetal genotypes are as follows: fetus 1, mosaic KO; fetus 3, mosaic KO; fetus 4, biallelic KO; fetus 5, biallelic KO.

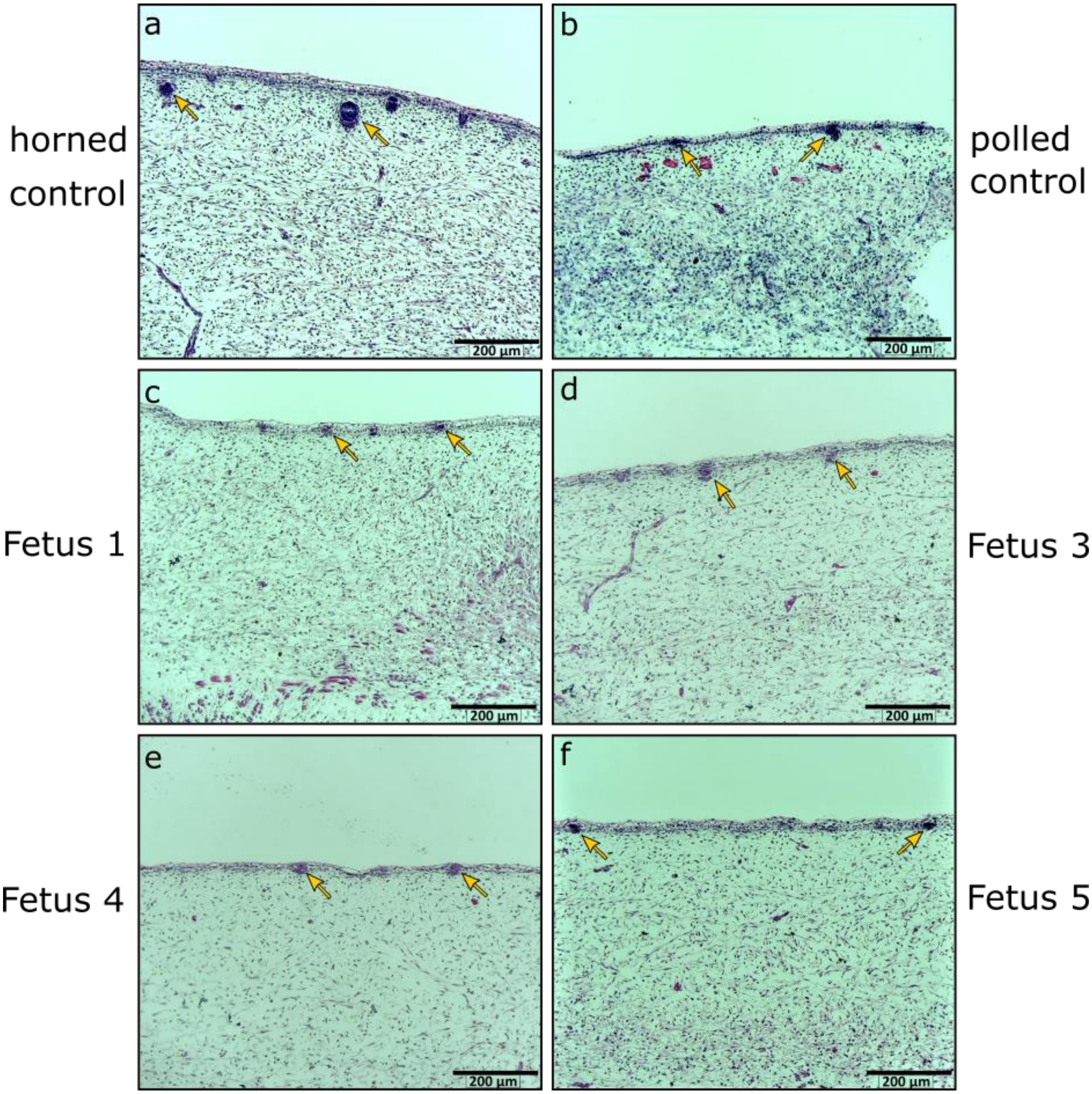

Supplementary Figure S3. Frontal skin histological analysis of 90-day fetuses harvested from embryo transfer (ET) 2 alongside horned and polled controls. (**a**) Fontal skin of horned and (b) polled age matched control fetuses alongside (c-f) the frontal skin of lincRNA#1 knockout (KO) fetuses at 90 days of gestation. Hair follicles can be seen in the frontal skin of all fetuses (yellow arrows). Stained with hematoxylin and eosin. Fetal genotypes are as follows: fetus 1, mosaic KO; fetus 3, mosaic KO; fetus 4, biallelic KO; fetus 5, biallelic KO.
